# Middle-Inner Macular Layers Dysfunction in a Case of Stellate Foveomacular Retinoschisis Detected by Abnormal Multifocal Photopic Negative Response Recordings

**DOI:** 10.3390/diagnostics12112753

**Published:** 2022-11-10

**Authors:** Lucilla Barbano, Giulio Antonelli, Mariacristina Parravano, Eliana Costanzo, Vincenzo Parisi, Lucia Ziccardi

**Affiliations:** IRCCS—Fondazione Bietti, Via Livenza 1, 00198 Rome, Italy

**Keywords:** SNIFR, PhNR, retinoschisis, inner retina, electroretinogram

## Abstract

We describe the macular morpho-functional assessment of a 65-year-old man affected by stellate nonhereditary idiopathic foveomacular retinoschisis (SNIFR), studied by visual field, SD-OCT, autofluorescence, full-field electroretinogram (ffERG), multifocal electroretinogram (mfERG) and multifocal Photopic Negative Response (mfPhNR) recordings. The typical presentation consists of the foveal appearance of radial cartwheel pattern for the splitting of the retinal layers at the level of the Henle fiber layer (HFL) and the outer plexiform layer (OPL), perfectly seen by Spectral Domain-Optical Coherence Tomography (SD-OCT). Despite a normal function of the outer retina of the peripheral and central retina evaluated by ffERG and mfERG respectively, we observed a reduced function of the retinal elements involved in the retinoschisis by recording mfPhNR that assesses mainly inner retina function (retinal ganglion cells and their axons). Therefore, it is likely that the observed impaired mfPhNR responses reflect the signaling defects derived from the delaminated middle retina and transmitted to the innermost retinal layers.

Stellate nonhereditary idiopathic foveomacular retinoschisis (SNIFR), firstly described by Ober et al. [1], is an uncommon retinal disorder characterized by foveal retinoschisis in the absence of inhered or acquired predisposing conditions such as X-linked retinoschisis, myopia, optic disc pit, glaucoma, myotonic dystrophy, enhanced S-cone syndrome, and vitreomacular tractions. SNIFR onset is described by the age of 60, it can be monolateral or bilateral and females are reported to be more affected than males [2]. The diagnosis is based on the typical appearance of the fovea presenting with the characteristic radial cartwheel pattern and with the splitting of the retinal layers at the level of the Henle fiber layer (HFL) and the outer plexiform layer (OPL) seen by Spectral Domain-Optical Coherence Tomography (SD-OCT). At the present, the SNIFR condition has been studied by multimodal imaging combining together SD-OCT [1,2], OCT angiography (OCTA), [3,4,5] fluorescin angiography (FA), [1] and multifocal electroretinogram (mfERG) [4]. However, there is a lack of information about the function of cellular elements located in middle-inner retinal layers, where the retinoschisis resides. A 65-year-old man (F.F) was referred to the Neurophtalmology Unit at IRCCS Fondazione Bietti in Rome for visual disturbances in both eyes (OU) in the past six months. Written informed consent was obtained from the patient to publish this paper. The patient mentioned normal visual acuity until the onset of the symptoms. No other ocular or systemic diseases nor family history for ophthalmological abnormalities were reported. Genetic testing was requested and resulted negative for the presence of gene mutations responsible for the main related retinal dystrophies (an NGS panel covering X-linked retinoschisis and enhanced S-cone syndrome). At our first observation, the patient’s best corrected visual acuity (BCVA), measured at 4 m, was 20 ETDRS letters in the right eye (RE) and 30 letters in the left eye (LE); the near vision was J10 characters in RE and J8 characters in LE, respectively. Color vision (assessed by Ishihara Charts) was abnormal in both eyes (OU: 1/22 in RE and 11/22 in LE) and the Amsler test was positive in OU. The anterior segment and intraocular pressure were within normal limits. The visual field exam (Humphrey 10-2 SITA standard program) showed relative central scotoma in OU, menacing the central vision. On fundus examination, we observed in the central retina, an altered foveal reflex with apparent foveal cysts in a stellate configuration without abnormalities in the mid and extreme periphery of the retina. Indeed, no peripheral schisis was present. SD-OCT scans and Autofluorescence (AF) images of the affected patient and of a representative control eye are shown in Figure 1.

Electro-functional studies were also performed on our patient. Dark- and light-adapted full-field electroretinograms (ffERG), 10 Hz and 30 Hz flicker ERGs (Retimax CSO, Firenze, Italy) have been recorded according to the International Society for Clinical Electrophysiology of Vision (ISCEV) standards [6,7]. Normal a- and b-wave amplitudes and implicit times have been recorded, thus showing normal whole retinal function in OU. In Figure 2 are displayed the ring analysis traces of outer and middle-inner retina functional responses, evaluated by mfERG and multifocal Photopic Negative Responses (mfPhNR), respectively.

In this case of SNIFR, we were able to establish the typical appearance of inner retina delamination by SD-OCT, as well as slight signs of morphological outer retina changes in the fovea (mild retinal pigmented epithelium abnormalities). These findings, however, were not associated to an impairment of the peripheral nor the central outer retina function. Indeed, dark- and light-adapted ffERG as well as 10 Hz and 30 Hz flicker ERGs recorded normal a- and b-wave amplitudes, and normal photoreceptors and bipolar cells function by mfERG was recorded from localized retinal areas of the macula. Our functional findings are in contrast with those reported by Yadav et al. [5], who described a case of SNIFR in a 64-year-old woman, but with concomitant diabetes and rheumatoid arthritis in treatment with hydrochloroquine therapy, reduced macular mfERG amplitudes and reduced amplitude of b-wave of scotopic ffERG. Since it is known that diabetes and the treatment with hydrochloroquine therapy might influence the macular function assessed by mfERG [10,11], in our opinion all that observed by Yadav et al. [5] cannot be ascribed exclusively to the retinal involvement due to the SNIFR. 

Although accurate multimodal imaging of retinas has been already performed by previous authors in SNIFR [1,2,3,4,5], there is a lack of information about the functional assessment of the middle-inner retina, involved in the structural damage. We thought the application of the novel technique of mfPhNR recordings that records mainly the function of retinal ganglion cells and their axons [12] from localized areas valuable. In fact, we recently described an application of assessment of the function of localized inner retinal areas by the innovative method of mfPhNR [9]. In PhNR signal genesis, a partial contribution could be given by glial cells too [13]. In the foveal area, the glia comprehends astrocytes and Müller cells that provide the structural stability of the foveal tissue and improve the light transmission through the layers to the photoreceptors [14]. 

Therefore, by recording mfPhNR in our case of SNIFR we found reduced RADs in all rings, likely reflecting the signaling defects arising from the delaminated middle retina to the innermost retinal layers, passing through the transmission of the intraretinal connection cells (Müller/glial cells). The application of this electrofunctional method allowed us to identify, for the first time, in this rare condition of SNIFR, the overall dysfunction of macular retinal ganglion cells along with their axons. This aspect, associated with an absence of outer retinal dysfunction (normal mfERG responses), could help us to make a differential diagnosis between SNIFR and other hereditary macular schisis [2,15]. The novel findings of middle-inner retinal macular dysfunction, detected by this not invasive electrophysiological method of mfPhNR analysis, give new insights into the still unknown pathogenesis of SNIFR. We acknowledge that this is only a single report; however, we believe that interesting conclusions can be drawn by applying this multimodal study to a larger number of similar patients.

## Figures and Tables

**Figure 1:**
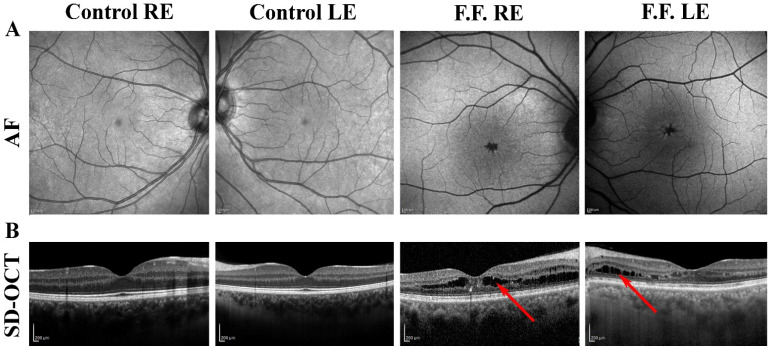
Morphological evaluation of the right eye (RE) and left eye (LE) of a patient affected by stellate nonhereditary idiopathic foveomacular retinoschisis (SNIFR) (F.F.), showed in comparison with a representative normal eye (Control eye). (**A**) Autofluorescence exam shows a foveal hypoautofluorescence with a radial aspect in F.F RE and LE. (**B**) Spectral Domain-Optical Coherence Tomography (SD-OCT, Heidelberg Engineering, Heidelberg, Germany) scan shows typical foveomacular schisis cavities at the level of Henle Fibers Layer (HFL) and Outer Plexiform Layer (OPL) (red arrows) associated with intraretinal hyperreflective foci and mild retinal pigmented epithelium abnormalities in F.F RE and F.F LE.

**Figure 2:**
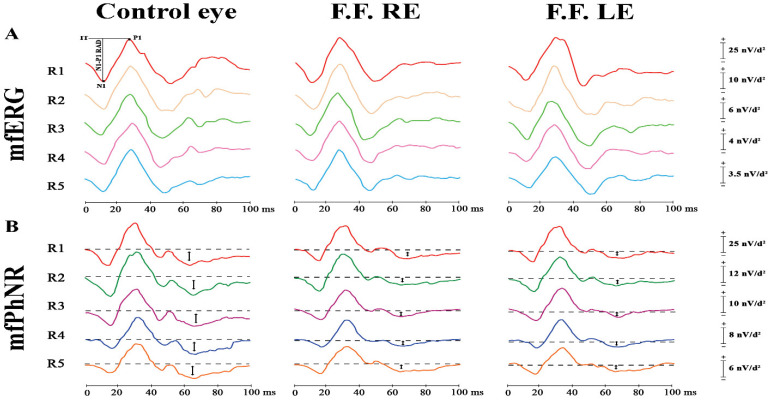
We assessed the function of the outer and middle-inner retina of our patient affected by stellate nonhereditary idiopathic foveomacular retinoschisis (SNIFR) (F.F.), whose results are compared in this figure with a representative normal eye (Control eye). (**A**) The preganglionic elements (photoreceptors and bipolar cells) of the macular region (from 0° to 25° centered to the fovea) were investigated by multifocal electroretinogram (mfERG: Diagnosys UK, LTD; Histon, Cambridge, UK) recordings. MfERG averaged responses were obtained from five concentric annular retinal regions (rings) centered on the fovea: from 0° to 5° (ring 1, R1 in red), from 5° to 10° (ring 2, R2 in yellow), from 10° to 15° (ring 3, R3 in green), from 15° to 20° (ring 4, R4 in purple) and from 20° to 25° (ring 5, R5 in blue). We considered the mfERG peak-to-peak Response Amplitude Density (RAD), measured in nanoVolt/degree^2^ (nV/deg^2^), between the first negative peak (N1) and the first positive peak (P1) and indicated by the arrow (↓) in five concentric rings (R) centered to the fovea with increasing foveal eccentricity. The values of N1-P1 RADs detected in all Rings of diseased eyes were within the normal limits that were extensively reported in our previously published paper [8]. Therefore, it can be observed that diseased eyes and control eyes showed similar traces. (**B**) We assessed also the macular function of localized middle-inner retinal layers containing ganglion cells and their axons and glial cells by measuring the RAD of the multifocal Photopic Negative Response (mfPhNR) (Diagnosys UK, LTD; Histon, Cambridge, UK) recordings. MfPhNR averaged responses obtained from rings 1 to 5 (R1 to R5, depicted by red, green, purple, blue and orange traces, respectively) are presented by measuring the RAD that is indicated by an arrow (↕). Reduced RADs were detected in all rings in OU as compared to our normal limits [9].

## Data Availability

Data available from authors upon request.
